# Pharmacogenetics of induction therapy-related toxicities in childhood acute lymphoblastic leukemia patients treated with UKALL 2003 protocol

**DOI:** 10.1038/s41598-021-03208-9

**Published:** 2021-12-09

**Authors:** Sara Aslam, Sonia Ameer, N. A. Shabana, Mehboob Ahmed

**Affiliations:** Department of Microbiology and Molecular Genetics, University of the Punjab, Lahore, 54590 Pakistan

**Keywords:** Cancer, Genetics, Molecular biology

## Abstract

Chemotherapy related toxicities have been the major factor limiting the success of acute lymphoblastic leukemia (ALL) induction therapy. Several factors, including the pharmacogenetics of asparaginase and anthracyclines, could contribute to difference in treatment outcome in ALL. We investigated the significance of variations in genes involved in hepatic and cardiac toxicity in acute lymphoblastic leukemia (ALL). Genotyping of *SOD2* (rs4880), *PNPL3* (rs738409) and *ABCC1* (rs4148350), C*BR1* (rs9024) and *ABCG2* (rs2231142) was performed by Tetra-ARMS PCR-based technique to evaluate the genotype–phenotype correlation. Our results showed only minor allele G of *SOD2* rs4880 increase the risk of hepatic toxicity [OR 2.63 (1.42–4.84), *P* =  < 0.05] while minor alleles of other SNPs showed protective impact. However, the genetic contrast analysis showed a recessive form of *SOD2* rs4880 [OR 7.82 (3.86–15.85), *P* =  < 0.05] and *PNPLA3* I148M [OR 5.82 (3.43–9.87), *P* =  < 0.05] variants whereas dominant genotype of *ABCC1* rs4148350 [OR 2.52 (1.55–4.10), *P* =  < 0.05] significantly predisposes hepatotoxicity. Furthermore, heterozygous form of *ABCG2* rs2231142 [OR 5.25 (1.84–14.95), *P* =  < 0.05] and recessive genotype of 3′UTR variant *CBR1* rs9024 [OR 2.31 (1.31–4.07), *P* =  < 0.05] were strongly associated with cardiotoxicity. The information obtained from these genetic variations could offer biomarkers for individualization of therapeutic intervention in ALL.

## Introduction

Acute lymphoblastic leukemia (ALL) is the predominant childhood malignancy and accounts for 20.8% of all the reported childhood cancers in the Pakistani population in 2018^1^. The cure rate of ALL is approaching 90% due to the significant advancements in understanding the biological heterogeneity of the disease, implementation of the risk-adapted therapies and effective chemotherapy treatment^2^. The combinations of asparaginase, vincristine, dexamethasone and anthracyclines have been the main induction regimens to treat ALL for decades. Complete remission rate of this induction therapy in ALL is reported more than 95%^3^ however, at the cost of significant life‐threatening toxicities^4^. Despite the great progress in curing ALL, the acute and chronic adverse effects of chemotherapy are the major challenges that impair survivor’s quality of life and prognosis of the disease. Among these, asparaginase allergy is most reported in pediatric ALL while hepatotoxicity is reported in adults with ALL^5^, whereas anthracyclines increase the risk of cardiotoxicity in both pediatric and adults with ALL^6^. The Pharmacogenetic studies focused on exploring hepatotoxicity and cardiotoxicity in pediatric ALL are still limited.

The mechanism of action of asparaginase related hepatotoxicity includes the Superoxide dismutase (SOD2), PNPL3 enzymes and an efflux drug transporter pump (ABCC1). Asparaginase induces the amino acid stress response that causes depletion of the asparagine and glutamine in the cells resulting in the excessive production of reactive oxygen species (ROS) and cell apoptosis. Superoxide dismutase (SOD) enzyme protects the cells by catalysing the ROS into oxygen and hydrogen peroxide. Previous studies have reported the *SOD2* variant (rs4880) causing the substitution of V16A amino acids to be significantly associated with drug-induced hepatotoxicity^7,8^. The genome-wide association study showed the significant association of *PNPLA3* rs738409 (C>G) I148M variant with the elevation of alanine transaminase (ATL) after the completion of induction therapy^9^. A study conducted in myeloid leukemia (AML) patients associates *ABCC1* (drug efflux pump) rs4148350 (G>T) variant with the severe hepatotoxicity^10^. The information on the mechanism of action of *PNPL3* and *ABCC1* in causing hepatotoxicity is still underreported.

Drugs metabolizing enzyme (CBR1) and the drug transporter pump (ABCG2) of the ATP-binding cassette (ABC) family are involved in causing anthracycline related cardiotoxicity. Carbonyl reductase 1 (CBR1) enzyme has been reported to convert anthracycline to their cardiotoxic metabolites and its overexpression is predominantly associated with cardiotoxicity and resistance to treatment^11^. However, the results of the meta-analysis showed the protective impact of the CBR1 (G>A) rs9024 variant^12^. The efflux drug transporter ABCG2 (G>A) rs2231142 variant has also been strongly associated with cardiotoxicity by a previous study conducted in acute myeloid leukeima patients (AML)^10^.

The current study is designed to elucidate the role of the *SOD2* (rs4880), *PNPL3* (rs738409) and *ABCC1* (rs4148350) variants with asparaginase related hepatotoxicity and *CBR1* (rs9024) and *ABCG2* (rs2231142) variants with anthracycline related cardiotoxicity. We anticipate that the information from the genetic variations will help in developing personalized medicine for acute lymphoblastic leukeima. The individualization of therapeutic intervention will contribute to reducing acute and chronic chemotherapy toxicities while identifying the patients who are most likely to benefit from the therapy, thereby improving the survival rate.

## Materials and methods

### Study population

This is a cross-sectional study that includes the toxicity profile of acute lymphoblastic leukemia patients (ALL) obtained retrospectively. A total of 300 pediatric ALL patients were recruited from the Children's Hospital and the Institute of Child Health, Lahore diagnosed from December 2018 to September 2019 and treated according to the UKALL 2003 protocol. The eligibility criteria for recruitment include the patient aged ≥ 15 years and diagnosed with ALL as primary cancer. The exclusion criteria include any secondary cancer, patient seropositive with infectious diseases or underlying cardiac disease before the start of treatment. The research was approved by the Research Ethics and Biosafety Committee at University of the Punjab (Reference number: SBS/222/18) and the ethical standards and protocols for data collection follow the national and Helsinki Declaration. A written informed consent was obtained from the legal guardians as patients were younger than ≥ 15 years.

### Treatment plan

The patients were stratified according to the UKALL 2003 protocol into NCI standard-risk group and NCI high-risk group. The standard-risk group constitute patients with B cell precursor ALL (BCP ALL) aged ≥ 1 year at diagnosis and < 10 and < 50 × 109/L WBC count at the start of treatment. The high-risk group includes the B cell precursor ALL (BCP ALL) aged ≥ 10 years at diagnosis and ≥ 50 × 109/L WBC count at the start of treatment. T cell ALL was independent of the risk-group however receives high risk induction treatment. The induction regimen for the standard-risk group includes three major drugs dexamethasone, vincristine and asparaginase (regimen A induction) while high-risk group were administered additional anthracycline (daunorubicin) (regimen B induction).

### Data collection

A self-reporting questionnaire was used for the data collection and standardized face to face interviews were conducted with patient’s guardians. The information collected from the interview includes the age and gender of the patient. Patient medical files were consulted to obtain data related to the risk group of patients, the clinical history of the patient, starting dates of induction treatment, ultrasound, and echocardiogram reports. Ultrasound of the patients exposed with asparaginase (both A and B regimen), and the echocardiogram of patients exposed with anthracycline (regimen B) was considered 15 days after remission induction. To evaluate the cardiotoxicity, the American Society of Echocardiography guidelines were considered that include Simpson method (biplane method of disks) for the assessment of any drop in LVEF > 10 to < 53%, presence of pericardial effusion, high muscular restrictive VSD, spontaneous closure of ASD, regressed pulmonary hypertension or tamponade present. To evaluate hepatotoxicity, the presence of hepatosplenomegaly or hepatomegaly (evaluated by comparing the increase in liver size by age with standard size) were considered.

### Genotyping

A millilitre (1 ml) of the venous blood samples of the 300 acute lymphoblastic leukemia (BCP ALL and T cell ALL) patients 15 days after remission induction were collected in the K_3_EDTA-coated tubes. The DNA extraction from blood samples patients was done according to the instructions of Sam brook 2001 organic protocol^13^ and stored at − 20 °C until used. The SNPs were screened from dbSNP^14^ and their presence in the Pakistani population was confirmed by the Ensemble genome browser^15^. To examine the polymorphism, the Tetra-ARMS primers were designed by using PRIMER 1 online software (http://primer1.soton.ac.uk/primer1.html)^16^ (Supplementary Table S1) and amplification was performed by using PCR thermal cycler (Bio-Rad^®^, USA) following gel electrophoresis.

### Statistical analysis

SPSS software version 22 and SNPStats (an online tool for SNP analysis)^17^ were utilized for the data analysis. Categorical data were presented in the form of percentages (%) and counts (N) and continuous variables were presented as mean ± SD. Allele and genotype frequency was calculated and compared by using two-tailed chi-square or Fisher exact tests as appropriate. Univariate linear regression was used to find the adjusted associations between homozygous major and minor genotypes with toxicity outcome. The statistical power of the study to detect association was considered 90% or above. To the increase the statistical power, a large sample size that is 4.3 times of the sample size calculated by keeping 4.7% leukemia cases in overall population (Globocan 2020) and the significance level for the associations was kept < 0.05. Bonferroni correction for multiple testing was applied for allele and genotype analysis.

### Ethics approval

The research was conducted accordance with the Declaration of Helsinki. The study was conducted at the University of the Punjab and the Research Ethics and Biosafety Committee of University of the Punjab approved the study protocol related to the data collection from the human patients. Informed written consent from the guardian of patients was obtained to enrol the patients in study since age of patients was < 16 years.

## Results

### Patient population and toxicity data

The present study analysed 300 samples of acute lymphoblastic leukemia patients. The mean age of the children at the diagnosis was 6.62 ± 3.5 years; the mean age of males was 6.80 ± 3.56 and females was 6.14 ± 3.34 years. The number of males 217 (72.33%) were more than the females is 83 (27.66%). The most common immunophenotype was BCP ALL (N = 259) following T cell ALL (N = 41). Our data showed the standard-risk group patients administered with induction regimen A were (N = 84) less than the high-risk group patients administered with induction regimen B were (N = 216). The hepatic toxicity profile of all the patients was analysed and hepatic toxicity (hepatosplenomegaly) was manifested in 108 (36%) patients 15 days after the remission induction. Among the high-risk group patients, 79 (36.57%) patients developed cardiotoxicity 15 days after the remission induction. It should be noticed that the underlying cardiotoxicity manifested, as 21(9.70%) patients developed pericardial effusion, 46 (21.29%) had drop-in baseline left ventricular ejection fraction (LVEF) and 10 (4.62%) had both pericardial effusion and drop in LVEF. A few patients 1 (0.46%) manifested high muscular restrictive VSD, spontaneous closure of ASD, regressed pulmonary hypertension and early sign of tamponade present, massive pleural effusion as described in (Supplementary Table S2). The drop-in baseline left ventricular ejection fraction (LVEF) > 10%to ⩽ 50% was reported 36% that is more than reported in our study and it may be attributed to the short follow up of patients in our study^18^. The distribution of the toxicities among age of patients, WBC count at the time of diagnosis, risk group of patients and types of ALL showed no significant association (Supplementary Table S3).

### Allele and genotype frequencies

Allele frequencies for hepatotoxicity assessment are described in (Supplementary Table S4) and genotype frequencies in Table 1. The minor allele frequency (MAF) in the patients with hepatotoxicity for V16A (rs4880), rs738409 C>G, rs4148350 G>T, were 44%, 37% and 45% respectively. The minor allele frequency of V16A (rs4880) was significantly associated with hepatotoxicity (OR 2.63, 95%CI 1.42–4.84, *P* =  < 0.05), however; rs738409 C>G and rs4148350 G>T showed protective impact as (OR < 1). The most common genotype in SOD2 V16A (rs4880) and ABCC1 rs4148350 G>T was homozygous dominant as AA (46.30%) and GG (45.40%) respectively, while in PNPLA3 rs738409 C>G was homozygous recessive GG (56.50%). Allele frequencies for cardiotoxicity assessment are described in (Supplementary Table S5) and genotype frequencies in Table 2. The minor allele frequency (MAF) in the patients with cardiotoxicity for rs9024 G>A, and rs2231142 G>T, were 33% and 46% respectively. The minor alleles of both rs9024 G>A (OR 0.55, 95% CI 0.31–0.98, *P* =  < 0.4) and rs2231142 G>T (OR 0.29, 95% CI 0.16–0.54, *P* =  < 0.05) showed protective impact on cardiotoxicity. The most common genotype in rs9024 G>A was homozygous recessive AA (60.80%) and in rs2231142 G>T was homozygous dominant GG (60.80). Results remined the same after applying the Bonferroni corrections.

**Table 1:** Genetic contrast model for hepatotoxicity in ALL patients.

Gene/SNP	Model	Genotype	Non-hepatotoxicity (%)	Hepatotoxicity (%)	OR (95% CI)	*P* value	*P* value*	AIC
*SOD2/*rs4880	Codominant	AA	60.40	46.30	1.00	< 0.05*	< 0.05*	358.70
		AG	33.33	19.40	0.76 (0.42–1.38)			
		GG	6.20	34.30	7.15 (3.44–14.85)			
	Dominant	AA	60.40	46.30	1.00	< 0.05*	< 0.05*	390.50
		GG/AG	39.60	53.70	1.77 (1.10–2.85)			
	Recessive	AA/AG	93.80	65.70	1.00	< 0.05*	< 0.05*	357.60*
		GG	6.20	34.30	7.82 (3.86–15.85)			
*PNPLA3/rs7 *38409	Codominant	CC	61.50	29.60	1.00	< 0.05*	< 0.05*	351.30
		CG	20.30	13.90	1.42 (0.70–2.89)			
		GG	18.20	56.50	6.43 (3.63–11.37)			
	Dominant	CC	61.50	29.60	1.00	< 0.05*	< 0.05*	367.40
		CG/GG	38.50	70.40	3.79 (2.29–6.28)			
	Recessive	CC/CG	81.80	43.50	1.00	< 0.05*	< 0.05*	350.20*
		GG	18.20	56.50	5.82 (3.43–9.87)			
*ABCC1/rs41 *48350	Codominant	GG	67.70	45.40	1.00	< 0.05*	< 0.05*	383.40
		GT	16.70	31.50	2.82 (1.57–5.05)			
		TT	15.60	23.10	2.21 (1.18–4.13)			
	Dominant	GG	67.70	45.40	1.00	< 0.05*	< 0.05*	381.80*
		TT/GT	32.30	54.60	2.52 (1.55–4.10)			
	Recessive	GG/GT	83.30	68.50	1.00	0.11	< 0.05*	393.50
		TT	15.60	23.10	1.63 (0.90–2.94)			

*P* value* (Bonferroni corrected) < 0.05 was considered significant.

**Table 2:** Genetic contrast model for cardiotoxicity in ALL patients (* shows significant values).

Gene/SNP	Model	Genotype	Non-cardiotoxicity (%)	Cardiotoxicity (%)	OR (95% CI)	*P* value	*P* value*	AIC
*CBR1/*rs9024	Codominant	GG	34.30	26.60	1.00	< 0.05*	< 0.05*	280.10
		GA	25.60	12.70	0.64 (0.27–1.53)			
		AA	40.10	60.80	1.95 (1.03–3.72)			
	Dominant	GG	34.30	26.60	1.00	0.24	< 0.05*	286.30
		AA/GA	65.70	73.40	1.44 (0.78–2.66)			
	Recessive	GG/GA	59.90	39.20	1.00	< 0.05*	< 0.05*	279.10*
		AA	40.10	60.80	2.31 (1.31–4.07)			
*ABCG2*/rs2231 142	Codominant	GG	43.80	60.80	1.00	< 0.05*	< 0.05*	240.70*
		GA	3.60	26.60	5.25 (1.84–14.95)			
		AA	52.50	12.70	0.17 (0.08–0.37)			
	Dominant	GG	43.80	60.80	1.00	< 0.05*	< 0.05*	281.90
		AA/GA	56.20	39.20	0.50 (0.29–0.88)			
	Recessive	GG/GA	47.50	87.30	1.00	< 0.05*	< 0.05*	250.50
		AA	52.50	12.70	0.13 (0.06–0.28)			

*P* value* (Bonferroni corrected) < 0.05 was considered significant.

### Association of genotypes with toxicities

The genetic contrast model for hepatotoxicity assessment is described in Table 1. In the co-dominant model, genotypes were analysed independently keeping homozygous dominant as reference. The homozygous genotype (GG) of V16A (rs4880) was associated with increased risk of hepatotoxicity (OR 7.15, 95% CI 3.44–14.85) whereas; heterozygous genotype (GG) showed protective impact (OR 0.76, 95% CI 0.42–1.38) on hepatotoxicity. We found that both dominant (OR 1.77, 95% CI 1.10–2.85) and recessive (OR 7.82, 95% CI 3.86–15.85) genetic contrast models to be associated with the high risk of hepatotoxicity, however based on Akaike information criterion (AIC) the recessive model was considered as the best fit model for V16A (rs4880). For rs738409 C>G, the co-dominant model showed homozygous minor genotype (OR 6.43, 95% CI 3.63–11.37) causing high risk than heterozygous genotype (OR 1.42, 95% CI 0.70–2.89). Both dominant (OR 3.79, 95% CI 2.29–6.28) and recessive models (OR 5.82, 95% CI 3.43–9.87) were also associated with the high risk of hepatotoxicity; however, the best fit model considered was the recessive model. For rs4148350 G>T the co-dominant model showed association of both heterozygous (OR 2.82, 95% CI 1.57–5.05) and homozygous minor (OR 2.21, 95% CI 1.18–4.13) genotypes with hepatotoxicity. The dominant (OR 2.52, 95% CI 1.55–4.10) and recessive (OR 1.63, 95% CI 0.90–2.94) models also showed positive association with hepatotoxicity, however, based on AIC values the dominant model was considered as the best fit model (Table 1).

The co-dominant model for rs9024 G>A showed high risk causing homozygous recessive genotype 1.95 (1.03–3.72) whereas; heterozygous genotype (OR 0.64, 95% CI 0.27–1.53) showed protective impact on cardiotoxicity. Moreover, the dominant and recessive models also reflect association with the outcome of cardiotoxicity and the best fit model considered was recessive model. For rs2231142 G>T, only heterozygous genotypes were associated with cardiotoxicity in co-dominant model while dominant and recessive model showed protective impact on the cardiotoxicity. Based on the AIC value, the best fit model considered for rs2231142 G>T was co-dominant model (Table 2).

**Figure 1:**
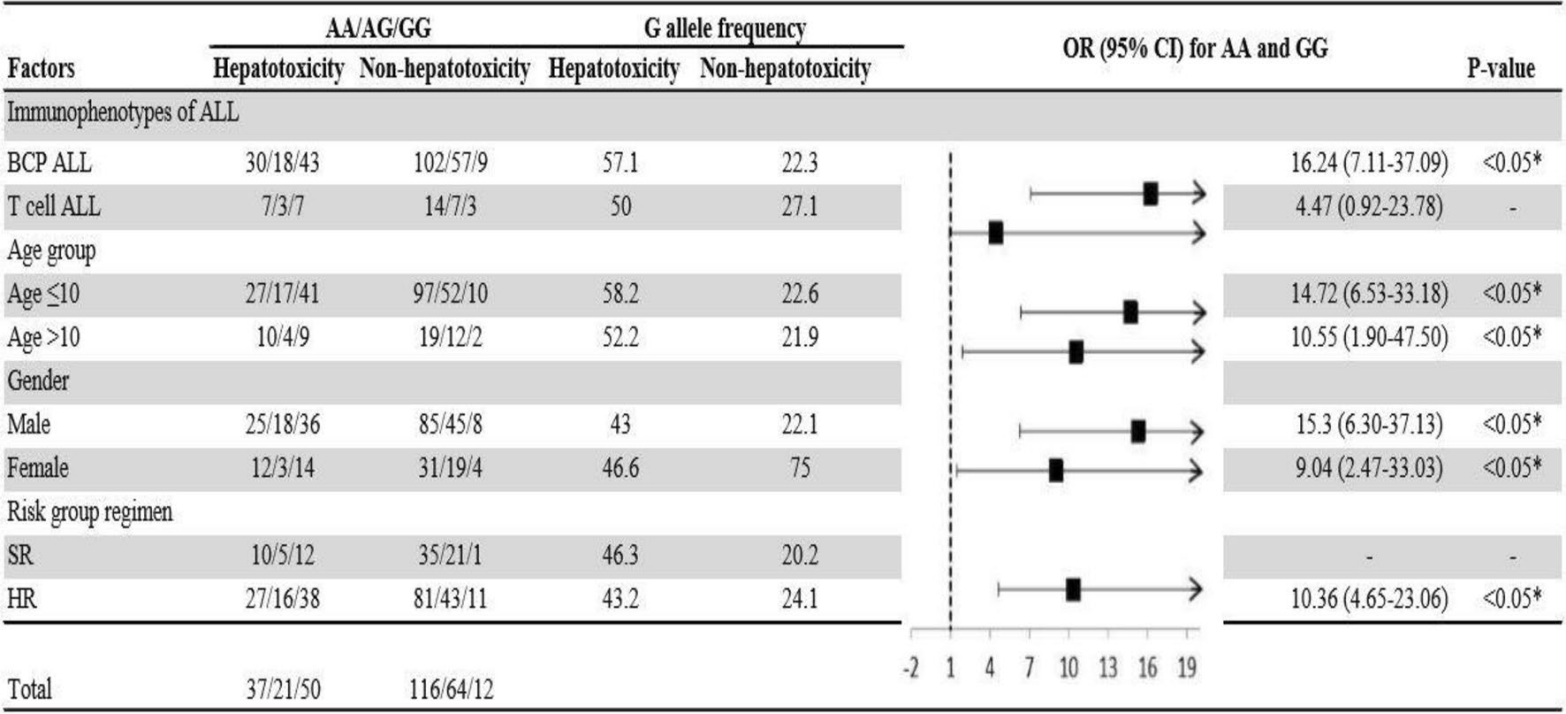
Adjusted association between *SOD2 *(rs4880) genotypes and risk of hepatotoxicity.

**Figure 2:**
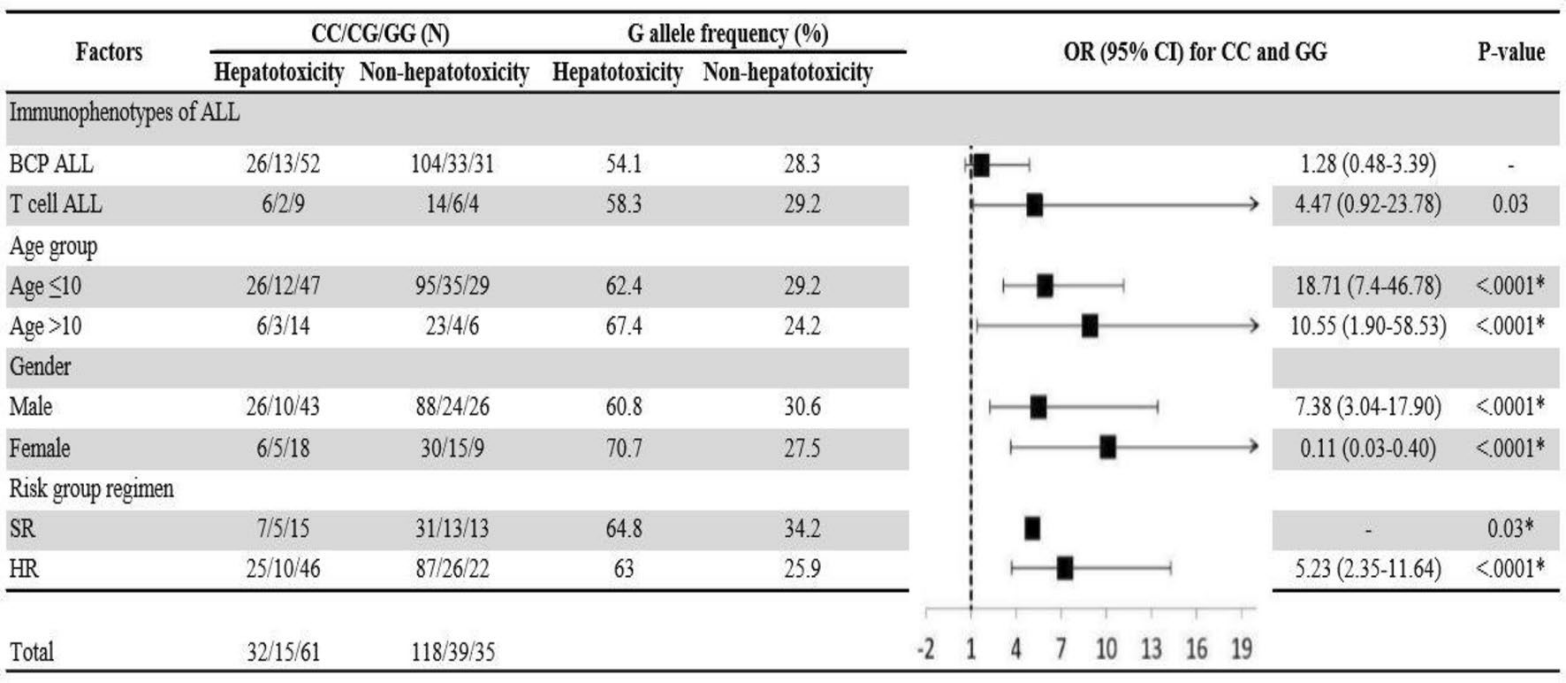
Adjusted association between *PNPLA3 *(rs738409) genotypes and risk of hepatotoxicity.

**Figure 3:**
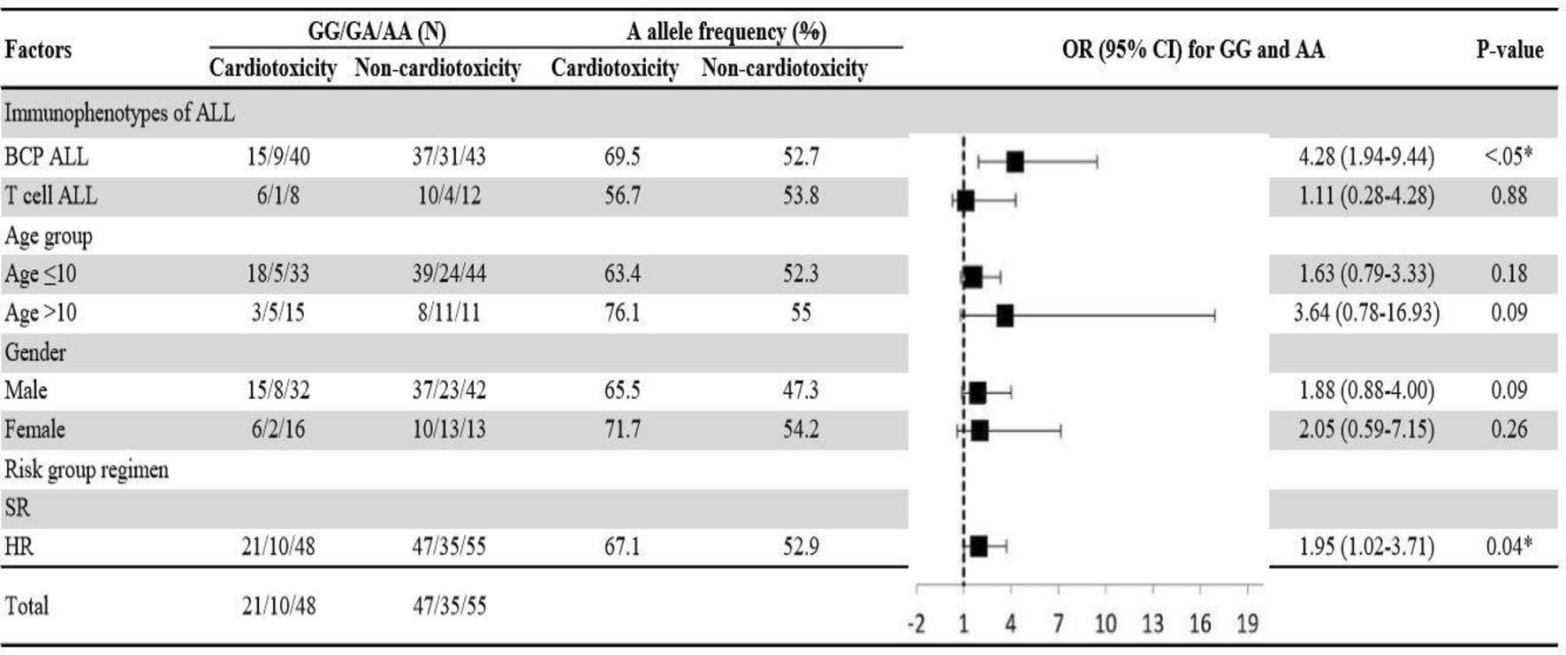
Adjusted association between *CBR1 *(rs9024) genotypes and risk of cardiotoxicity.

**Figure 4:**
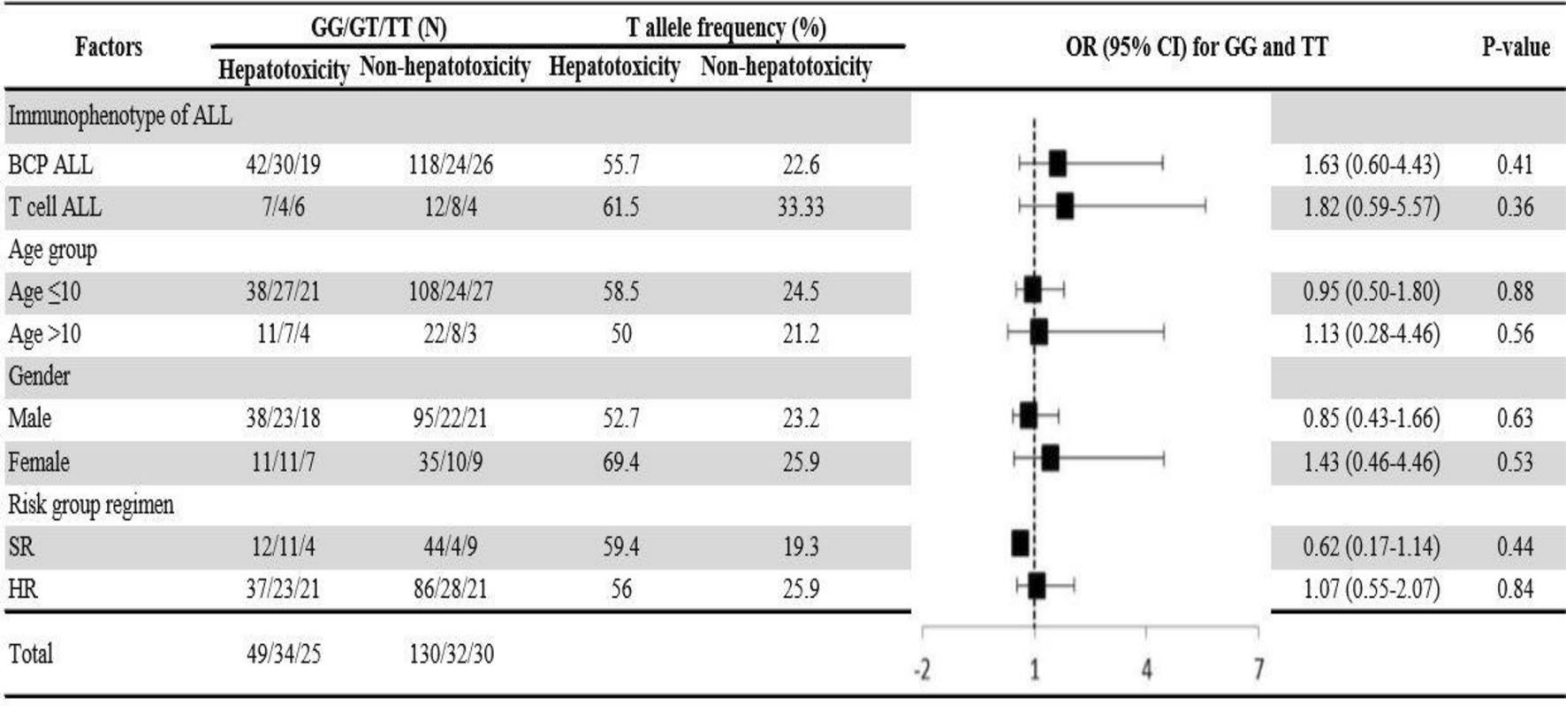
Adjusted association between *ABCC1 *(rs4148350) genotypes and risk of hepatotoxicity.

**Figure 5:**
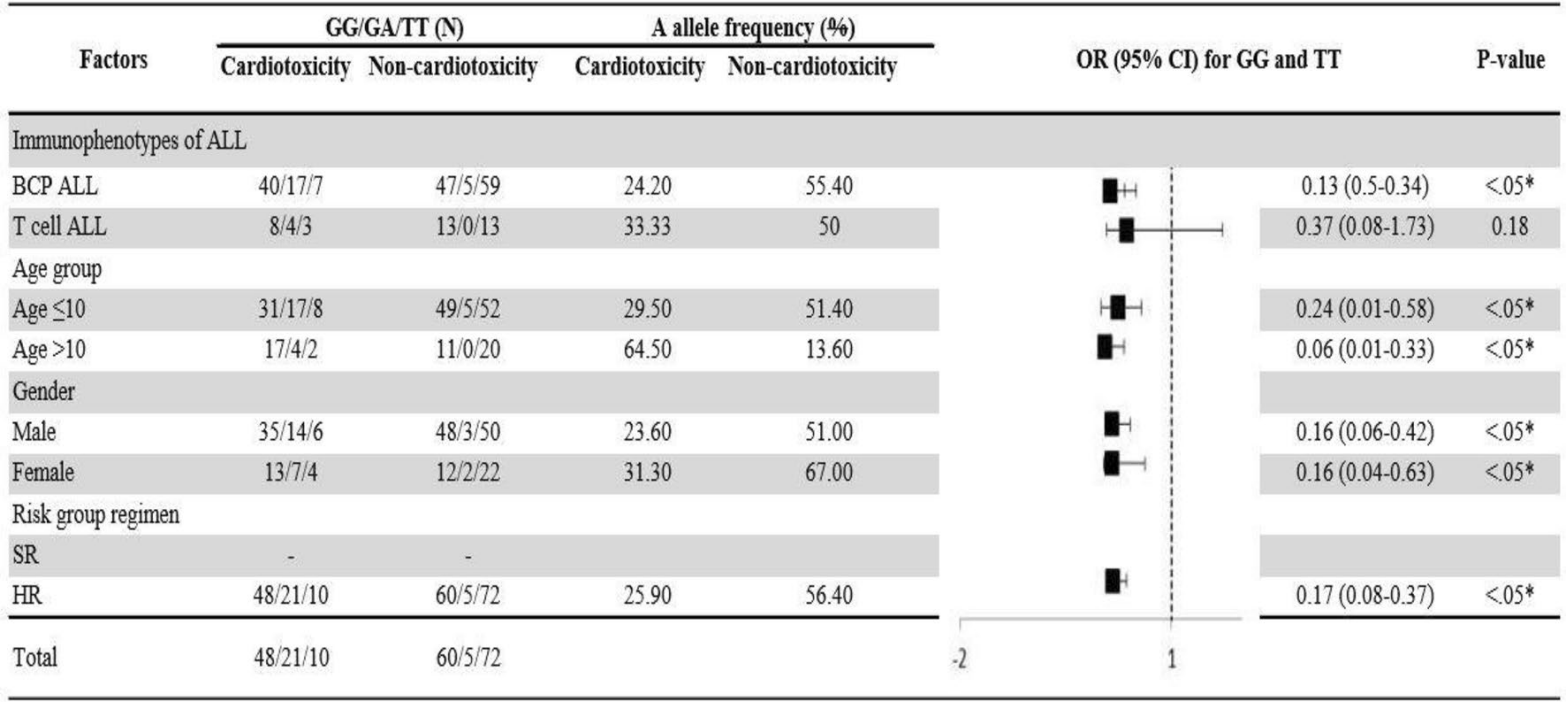
Adjusted association between *ABCG2 *(rs2231142) genotypes and risk of cardiotoxicity.

Adjusted association of the homozygous dominant and recessive genotypes with hepatotoxicity and cardiotoxicity were analysed by using univariate regression model. As shown in Figs. 1 and 2, BCP ALL male patients, aged ≤ 10 years on high-risk group regimen in the presence homozygous recessive allele of V16A (rs4880) and for rs738409 C>G T cell ALL male patients, aged ≤ 10 years on high-risk group regimen were at high risk of hepatotoxicity. Whereas the homozygous recessive genotype of rs9024 G>A was associated with high risk of cardiotoxicity in BCP ALL female patients aged > 10 years (Fig. 3). On contrary, rs4148350 G>T and rs2231142 G>T all the variables showed protective impact on hepatotoxicity and cardiotoxicity respectively (Figs. 4, 5). Therefore, it can be concluded that studied confounding variables impacts adverse outcome in the presence of SNPs.

## Discussion

The role of genetics in predisposition to adverse events to ASNase and anthracycline cardiotoxicity (ANTs) has been the focus of many previous studies. Most of the previous studies have analysed toxicities in adult patients, however the toxicity profile in pediatrics is less frequently investigated. Therefore, the aim of present study is to evaluate the effects of *SOD2, PNPLA3, ABCC1, CBR1* and *ABCG2* polymorphisms on ASNase and ANTs related toxicities in children with ALL. The key findings of this study suggest that polymorphisms in the studied genes could influence the toxicity of induction chemotherapy in pediatric ALL patients.

SOD2 is predominantly located in the mitochondrial matrix and plays a major role in the detoxification of reactive oxygen species^19^. The valine (T allele) form of SOD2 rs4880 results in faulty protein that partially traps in the inner mitochondrial membrane resulting in lower enzymatic efficiency and high likelihood of cancers and toxicities^7,20^. In our study, TT genotype in recessive genetic contrast model increases the risk of hepatotoxicity, in coincidence with the findings in adult Hispanic population^7^. The patients recruited by Alachkar et al., were also administered with other hepatotoxicity inducing drugs; methotrexate and 6-Mercaptopurine. Our study eliminates the limitation of their study as patients in our study were only administered with asparaginase and it affirms the role of SOD2 polymorphism in predisposition of hepatotoxicity.

Patatin-like Phospholipase Domain Containing Protein 3 (PNPLA3) is involved in metabolism and signalling of triacylglycerol. The substitution of the methionine at 148 position has been reported to significantly compromise the catalytic velocity in PNPLA3^21^. The knock-in mice studies also suggests that I148M variant contributes to the reduction in triglyceride hydrolase activity, and accumulation of hepatic triglycerides resulting in hepatotoxicity^22^. Our study reveals that GG genotype was associated with hepatotoxicity in a recessive genetic contrast model. In contrast with our study that found association of GG genotype with organ toxicity, a previous study assessed the role of I148M variant with serum hepatotoxicity: alanine transaminase (ALT) levels. The authors conducted a genome-wide approach in the cohort of racially diverse set of pediatric patients with ALL and found significant genetic association with elevation of ALT levels (*P* = 2.5 × 10^−8^) ^9^.

The impact of the drug transporter pump of ATP-binding cassette (ABC) family treatment efficacy has been well studied in various cancers. The results of our study showed the dominant genotype TT of rs4148350 G>T was associated with organ hepatotoxicity; in contrast with other study that found the strong association of heterozygous genotype GT with the severe hepatotoxicity (OR 5.3, CI 1.05–26.9, *P* = 0.04) in AML patients above 14 years^10^. Other studies found association of rs4148350 G>T with childhood cancer^23^, as well as febrile neutropenia in breast cancer^24^. A study in childhood leukemia patients associates low expression of mRNA of ABCC1 and ABCG2 with higher risk of toxic deaths^25^. ABCG2, a multidrug transporter functions for an efflux of anthracyclines from cardiomyocytes and blast cells. ABCG2 has been reported to play the pivotal role in cardiac repair after myocardial infarction^26^. Nevertheless, the rs2231142 G>T variant was identified for their ability to decrease expression of ABCG2 protein^27^, probably that reduces efflux of toxic metabolites of anthracyclines. Our study recorded greater cardiac toxicity with heterozygous genotype GA; in concordance with the study conducted in adult AML patients^10^.

CBR1 is an oxido-reductase enzyme that converts daunorubicin (Dnr) to DOL in ALL. Inhibiting over-expression of CBR1 is associated with increased efficacy of Dnr and reducing the risk of cardiotoxicity^28^. Our study showed a strong association of homozygous recessive genotype AA of 3′UTR variant rs9024 with cardiotoxicity, and these findings are in consistent with reports in Asian breast cancer patients relating the variant genotype with high systemic exposure and lower doxorubicin clearance^29^. The limitations of this study include the selection of candidate gene approach and its limited ability of replication of results, current follow-up still too short for further outcome analyses, and interference of other antibacterial and antifungal agents administered to the patients could contribute to the development of cardiotoxicity and hepatic toxicity. Moreover, impact of the analysed polymorphisms in clinical practice without providing functional data is very low. Despite these limitations, our findings underscore the predictive impact of genetic variability upon the toxic outcome in ALL induction therapy.

## Conclusion

In sum, our results suggest that genetic variants in the genes involved in hepatic and cardiotoxicity could influence the safety of standard induction treatment in pediatric acute lymphoblastic leukemia patients. The recessive form of *SOD2* rs4880 and *PNPLA3* I148M variants play role in predisposition of hepatotoxicity whereas dominant genotype of *ABCC1* rs4148350 was associated with hepatotoxicity. Furthermore, heterozygous form of *ABCG2* rs2231142 and recessive genotype of 3′UTR variant *CBR1* rs9024 were strongly associated with cardiotoxicity in ALL patients. To eliminate the limitations of present study, a larger prospective study with a long-term follow-up of the patients in the same ethnic group is needed to be conducted to identify genetic variants contributing the chronic or late adverse effects of induction therapy.

## Supplementary Information

Supplementary Tables.

## Data Availability

The datasets used and/or analysed during the current study are available from the corresponding author on reasonable request.
