# Supplementary material for: Pharmacogenetics of induction therapy-related toxicities in childhood acute lymphoblastic leukemia patients treated with UKALL 2003 protocol
Source: Sci Rep. 2021 Dec 9;11:23757. doi: 10.1038/s41598-021-03208-9 (PMC8660848; doi:10.1038/s41598-021-03208-9)
Supplement: Supplementary file 1 — Supplementary Tables. [file 41598_2021_3208_MOESM1_ESM.docx]

**Table 1:** Tetra-ARMS primers

| **Name** | **Sequence** | **T_m_** |
| --- | --- | --- |
| SOD2: G allele | AGGCAGCTGGCTCCGTTC | 60.8 |
| SOD2: A allele | GGAGCCCAGATACCCCCAA | 61.6 |
| SOD2: Forward outer  primer | TGGCGACAGTGGCCGCAC | 62.9 |
| SOD2: Reverse outer  primer | TCCTGGTACTTCTCCTCGGT | 60.5 |
| PNPLA3: G allele | CTTGGTATGTTCCTGCTTCTTG | 60.1 |
| PNPLA3: C allele | ATAAGGCCACTGTAGAAGCGG | 61.2 |
| PNPLA3: Forward outer  primer | CATGGATTAACCTACTCTGTGC | 60.1 |
| PNPLA3: Reverse outer  primer | TGTCTGAAAGGCAGTGAGGC | 60.5 |
| ABCC1: G allele | GCACAGAGGGTTCCCCGG | 62.9 |
| ABCC1: T allele | AAGGCTGCTGTAACTTACAACCA | 60.9 |
| ABCC1: Forward outer  primer | GGCTTTTGTTAAACGTGGATTCG | 60.9 |
| ABCC1: Reverse outer  primer | AGACCAGGAGAAAGTGAAACACA | 60.9 |
| CBR1: G allele | GATCTCTTATCAATTAGCACTCACTACTG | 59 |
| CBR1: A allele | AGTGCGTATTGCTCAATTAGTAGGAT | 60 |
| CBR1: Forward outer  primer | ATGGACAATTTGTTTCAGAGAAGA | 59 |
| CBR1: Reverse outer  primer | ACAAATGGACACTTATAAAGAACCATT | 59 |
| ABCG2: T allele | TCTGACGGTGAGAGAAAACTCAA | 60.9 |
| ABCG2: G allele | CCGAAGAGCTGCTGAGAATTG | 61.2 |
| ABCG2: Forward outer  primer | TATAGCAGGCTTTGCAGACATCTA | 62 |
| ABCG2: Reverse outer  primer | ATTTTATCCACACAGGGAAAGTCCTA | 62.9 |

**Table 2**: Characteristics of indexed patients

| **Characteristics** | **Number** | **Percentage (%)** |
| --- | --- | --- |
| Mean age at diagnosis ±SD | 6.62±3.5 | - |
| Mean age of males | 6.80±3.56 | - |
| Mean age of females | 6.14±3.34 | - |
| **Type of ALL** | | |
| BCP-ALL | 259 | 86.33 |
| Pre-T cell ALL | 41 | 13.66 |
| **Gender** | | |
| Male | 217 | 72.33 |
| Female | 83 | 27.66 |
| **WBC count (per mcL) at diagnosis** | | |
| <50,000 | **109** | 36.33 |
| ≥50,000 | **191** | 63.66 |
| **Bilirubin (mg/dL) after induction remission** | | |
| ≤0.2-1 | 217 | 72.33 |
| >1 | 83 | 27.66 |
| **Alanine transaminase (ALT) (U/L) after induction remission** | | |
| ≤35 | 152 | 50.66 |
| >35 | 148 | 49.33 |
| **Alkaline phosphatase (ALP) (U/L) after induction remission** | | |
| ≤350 | 247 | 82.33 |
| >350 | 53 | 17.66 |
| **Parental smoking** | | |
| YES | 118 | 39.33 |
| NO | 181 | 60.33 |
| **Administer risk group regimen** | | |
| SR regimen | 84 | 28.00 |
| HR regimen | 216 | 72.00 |
| **Hepatotoxicity (Total=300)** | | |
| Yes | 108 | 36.00 |
| No | 192 | 64.00 |
| **Cardiotoxicity in high-risk group (Total=216)** | | |
| No | 137 | 63.42 |
| Yes | 79 | 36.57 |
| Pericardial effusion | 21 | 9.70 |
| Drop in baseline left  ventricular ejection fraction (LVEF) | 46 | 21.29 |
| Both pericardial effusion and  drop in LVEF | 10 | 4.62 |
| High muscular restrictive VSD, spontaneous closure of ASD, regressed pulmonary hypertension | 1 | 0.46 |
| Moderate size pericardial effusion all around the heart, early sign of tamponade present, massive pleural effusion | 1 | 0.46 |

**Table 3:** Distribution of toxicities among demographic and clinical characteristics.

| **Characteristics** | **Hepatotoxicity (n= 108)**  **N (%)** | **No hepatotoxicity (n= 192)**  **N (%)** | **Odds ratio**  **(95% CI)** | **P-value** | **Cardiotoxicity (n= 79)**  **N (%)** | **No Cardiotoxicity (n= 137)**  **N (%)** | **Odds ratio**  **(95% CI)** | **P-value** |
| --- | --- | --- | --- | --- | --- | --- | --- | --- |
| **Age of the patient** | | | | | | | | |
| <10 | 83 (76.85) | 147 (76.56) | 1.01 (0.58-1.78) | 0.53 | 54 (68.35) | 95 (69.34) | 0.95 (0.52-1.73) | 0.49 |
| ≥10 | 25 (23.15) | 45 (23.44) |  |  | 25 (31.65) | 42 (30.66) |  |  |
| **WBC count at the time of diagnosis** | | | | | | | | |
| <50,000 | 39 (36.11) | 71 (36.97) | 0.96 (0.59-1.57) | 0.90 | 37 (46.84) | 48 (35.04) | 1.63 (0.92-2.87) | 0.11 |
| ≥50,000 | 69 (68.89) | 121 (63.02) |  |  | 42 (53.16) | 89 (64.96) |  |  |
| **Risk group** | | | | | | | | |
| Standard | 27 (25.00) | 57 (29.69) | 0.78 (0.46-1.34) | 0.42 | - | - | - | - |
| High | 81 (75.00) | 135 (70.31) |  |  | 79 (100) | 137 (100) |  |  |
| **Types of ALL** | | | | | | | | |
| BCP-ALL | 91 (84.26) | 168 (87.50) | 0.76 (0.39-1.49) | 0.48 | 64 (81.01) | 111 (81.02) | 0.99 (0.49-2.02) | 0.56 |
| Pre-T cell ALL | 17 (15.74) | 24 (12.50) |  |  | 15 (18.99) | 26 (18.98) |  |  |

**Table 4:** Association of allele frequency with hepatotoxicity in ALL patients

| **Gene/SNP ID** | **Allele/ genotype** | **Non- Hepatotoxicity**  **(%)** | **Hepatotoxicity (%)** | **OR (95% CI)** | **P-value** | **P-value*** |
| --- | --- | --- | --- | --- | --- | --- |
| **SOD2/rs4880** | G | 23 | 44 | 2.63 (1.42-  4.84) | <.05* | <.05* |
|  | A | 77 | 56 |  |  |  |
| **PNPLA3/rs738409** | C | 72 | 37 | 0.22 (0.12-  0.41) | <.05* | <.05* |
|  | G | 28 | 63 |  |  |  |
| **ABCC1/rs4148350** | G | 76 | 45 | 0.45 (0.23-  0.87) | 0.02* | <.05* |
|  | T | 24 | 31 |  |  |  |

P-value* (Bonferroni corrected)

<.05 was considered significant

**Table 5**: Association of allele frequency with cardiotoxicity in ALL patients.

| **Gene/SNP**  **ID** | **Allele/ genotype** | **Non-**  **cardiotoxicity (%)** | **Cardiotoxicity (%)** | **OR (95% CI)** | **P-value** | **P-value*** |
| --- | --- | --- | --- | --- | --- | --- |
| **CBR1/rs9024** | G | 47 | 33 | 0.55 (0.31-  0.98) | 0.04* | <.05* |
|  | A | 53 | 67 |  |  |  |
| **ABCG2/ rs2231142** | G | 74 | 46 | 0.29 (0.16-  0.54) | <.05* | <.05* |
|  | T | 26 | 54 |  |  |  |

P-value* (Bonferroni corrected)

<.05 was considered significant
